# A new, feasible, and convenient method based on semantic segmentation and deep learning for hemoglobin monitoring

**DOI:** 10.3389/fmed.2023.1151996

**Published:** 2023-08-03

**Authors:** Xiao-yan Hu, Yu-jie Li, Xin Shu, Ai-lin Song, Hao Liang, Yi-zhu Sun, Xian-feng Wu, Yong-shuai Li, Li-fang Tan, Zhi-yong Yang, Chun-yong Yang, Lin-quan Xu, Yu-wen Chen, Bin Yi

**Affiliations:** ^1^Department of Anesthesiology, Southwest Hospital, Third Military Medical University (Army Medical University), Chongqing, China; ^2^Chongqing Institute of Green and Intelligent Technology, Chinese Academy of Science, Chongqing, China

**Keywords:** continuous hemoglobin monitoring, deep learning, semantic segmentation, mask R-CNN, MobileNetV3

## Abstract

**Objective:**

Non-invasive methods for hemoglobin (Hb) monitoring can provide additional and relatively precise information between invasive measurements of Hb to help doctors' decision-making. We aimed to develop a new method for Hb monitoring based on mask R-CNN and MobileNetV3 with eye images as input.

**Methods:**

Surgical patients from our center were enrolled. After image acquisition and pre-processing, the eye images, the manually selected palpebral conjunctiva, and features extracted, respectively, from the two kinds of images were used as inputs. A combination of feature engineering and regression, solely MobileNetV3, and a combination of mask R-CNN and MobileNetV3 were applied for model development. The model's performance was evaluated using metrics such as R^2^, explained variance score (EVS), and mean absolute error (MAE).

**Results:**

A total of 1,065 original images were analyzed. The model's performance based on the combination of mask R-CNN and MobileNetV3 using the eye images achieved an R^2^, EVS, and MAE of 0.503 (95% CI, 0.499–0.507), 0.518 (95% CI, 0.515–0.522) and 1.6 g/dL (95% CI, 1.6–1.6 g/dL), which was similar to that based on MobileNetV3 using the manually selected palpebral conjunctiva images (R^2^: 0.509, EVS:0.516, MAE:1.6 g/dL).

**Conclusion:**

We developed a new and automatic method for Hb monitoring to help medical staffs' decision-making with high efficiency, especially in cases of disaster rescue, casualty transport, and so on.

## 1. Introduction

Continuous monitoring of hemoglobin (Hb) helps doctors make better decisions regarding blood transfusions. The most frequently used methods for Hb monitoring are automatic blood analysis and arterial blood gas (ABG) analysis, which require professional operators and devices. Therefore, they are not ideal for continuous Hb monitoring, especially during disaster rescue scenes, field rescue, emergent public health events (e.g., COVID-19), casualty transport, and battlefield rescue. Pulse co-oximetry hemoglobin (SpHb) was developed by Masimo Corporation, which is continuous and non-invasive and is used for providing additional and relatively precise information between measurements of Hb by invasive blood samples. However, its accuracy depends on the blood flow and temperature of the tested fingers (1). Additionally, the SpHb cannot be used with other monitors, thus restricting its clinical application.

Recently, non-invasive methods for continuous Hb monitoring based on computer vision technology have shown great potential (Supplementary Table 1). The basis of these methods is that the palpebral conjunctiva and the nailbed pallor could be used to diagnose anemia (2). Most of the studies focused on using the image of the palpebral conjunctiva to detect anemia. The typical characteristics of research in this area were as follows: first, images were obtained using special devices (fundus cope or macro-lens) (3–6) or consumer-grade smartphones or cameras (7, 8), among which models based on images obtained by fundus cope achieved the best performance (with an R^2^ value of 0.52, and area under the receiver operating characteristic curve (AUROC) of 0.93) (6); second, instead of estimating the exact concentration of Hb, detecting anemia patients was more common (5, 9–11), which may be associated with the small sample size of images (Supplementary Table 1); third, most of the model inputs were features extracted from the manually selected palpebral conjunctiva (7, 12); however, recently semantic segmentation algorithms were also applied to realize automatic estimation (3, 4, 13).

Above all, new methods for continuous Hb monitoring with the three advantages are badly needed: no requirement for a special device or position during image acquisition, automation presented by using eye images as model input; the ability to estimate the exact concentration of Hb; and the ability to detect anemia with different thresholds. Therefore, we aimed to develop a new method that combines semantic segmentation and deep learning algorithms to estimate the exact concentration of Hb for surgical patients with the eye images obtained using smartphones, to compare the model's performance with models based on feature engineering and solely deep learning methods using the eye and manually selected palpebral conjunctiva images, respectively, and to find out whether it would be promising for clinical and special situations.

## 2. Materials and methods

The study protocol was approved by the institutional ethics committee of the First Affiliated Hospital of the Third Military Medical University (also called Army Medical University, KY2021060) on February 20, 2021, and written informed consent was obtained from each patient. The clinical trial was registered on the Chinese Clinical Trial Registry (No. ChiCTR2100044138) on March 11, 2021. The principal researcher was Prof. Bin Yi. Patient enrollment and image acquisition were completed at the First Affiliated Hospital of the Third Military Medical University in Chongqing, China, between March 18, 2021, and April 26, 2021.

### 2.1. Patient enrollment and image acquisition

The inclusion criteria were as follows: volunteering to participate in the research; ABG analysis needed according to routine clinical practice; Hb variance larger than 1.5 g/dL perioperatively. The exclusion criteria were as follows: suffering eye diseases, eye irradiation, or receiving facial radiation therapy, suffering carbon monoxide poisoning, nitrite poisoning, jaundice, or other systemic diseases that would change the color of the palpebral conjunctiva.

There were eight researchers who participated in the research: one for patient enrollment, two for image acquisition, two for data collection and collation, one for palpebral conjunctiva identification, and two for quality control. One day before the operation, all patients who met the criteria and were willing to participate in the study signed written informed consent. On the surgical day, when the enrolled patients were undergoing ABG analysis, two researchers came to the operation room or the post-anesthetic care unit (PACU) to take pictures of the right and left faces with the standard exposing way of the palpebral conjunctiva in the routine light of the operation room and PACU. The time between ABG analysis and image acquisition was within 10 min. All the images were obtained when patients were in a supine position and by the rear camera of the same smartphone (20.00 megapixels and f/1.8 aperture) with the same parameters. At the same time, the other two researchers collected patients' information. After the whole day of image acquisition, the two researchers, for data collection and collation, picked out images obtained from the patients whose Hb variation was larger than 1.5 g/dL. The unselected images were all deleted permanently. The selected half-face images were cut as eye images following the criteria shown in Supplementary Figure 1. During the whole process, the two researchers for quality control checked the enrollment, images, basic information, and so on.

### 2.2. Image pre-processing

**Figure 1:**
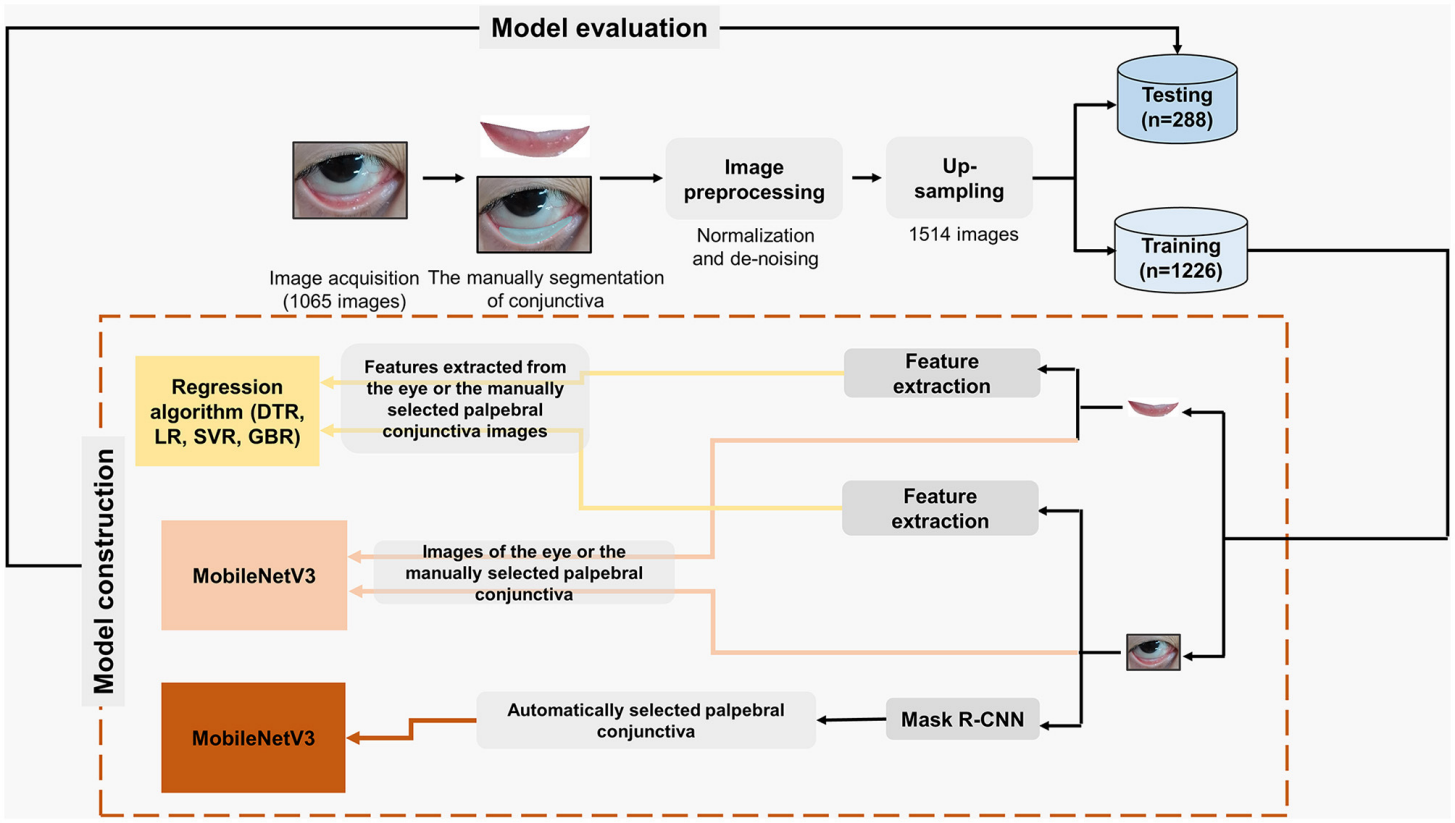
Flow diagram for estimating the exact concentration of Hb based on different algorithms with different inputs. DTR, Decision Tree regression; LR, Linear Regression; SVR, Support Vector regression; GBR, Gradient Boosting regression; CNN, Convolutional Neural Network.

As shown in Figure 1, after image acquisition, manual palpebral conjunctiva recognition, image pre-processing, and up-sampling were conducted. To keep the same standard of palpebral conjunctiva identification, one researcher worked on the manual segmentation of palpebral conjunctiva via Photoshop (Photoshop cs 6.0, Adobe Systems, California, USA) and Colabeler (version 2.0.4, Hangzhou Kuaiyi Technology Co. Ltd., Hangzhou, China). Subsequently, the eye and the palpebral conjunctiva images by Photoshop and Colabeler were normalized to a fixed size (500 × 500) to avoid possible loss of useful information as previously described (7). Due to that, different shapes and sizes of bright spots on the images were unavoidable, and denoising was also conducted. In the current study, K-means clustering was applied to identify the bright spot area in the Gray-level image converted from a corresponding RGB color image, and then the values of all pixels in the bright spot area were replaced by the mean value of all pixels in the non-bright spot area as previously described (7).

### 2.3. Feature extraction for the eye and the palpebral conjunctiva images

As shown in Figure 1, features extracted from the eye and the palpebral conjunctiva images were inputted for regression. The methods for feature extraction from the palpebral conjunctiva were relatively mature, so we applied the same algorithm for feature extraction as the study conducted by Miaou et al. (7). However, in the current study, we utilized normalized eye images and the palpebral conjunctiva as inputs for feature extraction, rather than relying on a manually selected fixed rectangular area. We extracted 18 features, including Hue Ratio, Pixel Values in the Middle, Entropy H to describe the distribution of the blood vessels and Binarization of the High Hue Ratio.

#### 2.3.1. Automatically segmentation of the palpebral conjunctiva by mask R-CNN

Herein, automatic recognition of the palpebral conjunctiva from eye images was achieved using mask R-CNN (14). Mask R-CNN is an instance segmentation framework extended by Faster RCNN (15), which could simultaneously perform pixel-level object segmentation and target recognition. It operates in two stages: the first stage scans the image and generates suggestions, and the second stage classifies the suggestions, generates bounding boxes, creates masks for accurate delineation of the recognized objects. Except for the original Faster RCNN network structure, the mask R-CNN also included the feature pyramid network (16) and the region of interest alignment algorithm (ROI Align) (14). Detailed information is given in the Supplementary Methods and Supplementary Figure 2. For semantic segmentation performance. We report the average precision (AP) and average recall (AR) over mask Intersection-over-Union (mIoU) thresholds (50%, 75%, 50%, and 95%). The segmentation work was conducted using ubuntu16.04TSL, Pytorch 1.3, and CUDA 11.0 platforms.

### 2.4. Establishment of models for the exact concentration of Hb based on different algorithms

As shown in Figure 1, all the extracted features were inputted to develop models. Models were fitted with decision tree regression, linear regression, support vector regression, and gradient boosting regression, respectively. MobileNetV3 (17) was applied to models directly using the eye and the palpebral conjunctiva images. In the current study, the classification structure of the mobilenetV3 tail was changed to a regression structure for the exact concentration of Hb. The mean square error loss function was used for training. These experiments used the open-source PyTorch learning framework and Python programming to realize the algorithm network. The hardware environment is a Dawning workstation from Chongqing Institute of Green and Intelligent Technology, Chinese Academy of Sciences, equipped with dual NVIDIA 2080Ti graphics cards (11 GB) and a 64-bit Ubuntu16.04 operating system (detailed information is shown in Supplementary Methods and Supplementary Figure 2).

### 2.5. Establishment of models based on the combination of mask R-CNN and MobileNetV3

We attempted to estimate the exact concentration of Hb based on the mask R-CNN and MobileNetV3 in two steps: semantic segmentation and regression (Supplementary Figure 2). First, semantic segmentation was performed to automatically recognize the palpebral conjunctiva from the eye images. Then, the recognized palpebral conjunctiva images were entered into the MobileNetV3 network to estimate the exact concentration of Hb. This two-step method could automatically estimate the exact concentration of Hb with eye images.

### 2.6. Model evaluation

For estimating the exact concentration of Hb, we evaluated the model's performance with the mean absolute error (MAE), R^2^ and Explained variance score (EVS). The MAE is used to describe the average difference between the estimated value and the actual value. The EVS describes the similarity between the dispersion degree of the difference between all predicted values and samples. EVS was calculated by the following formula: $EVS\left(y,ŷ\right)=1-\frac{Var\left\{y-\;ŷ\right\}}{Var\left\{y\right\}}$, where *y* is the Hb measured by ABG analysis, ŷ is the estimated Hb, and Var is the square of the standard deviation. R^2^ is also called the coefficient of determination. The closer the value to 1, the stronger the ability to interpret the output and the better the model fitting. Furthermore, we paid more attention to whether the new method could provide a relatively precise trend of Hb and recognize anemia with different thresholds. In addition to evaluating the model's performance using regression parameters, we investigated the correlation between the estimated and actual Hb and the ability to recognize anemia patients (Hb <10.0 g/dL, 11.0 g/dL, and 12.0 g/dL) according to the estimated Hb. Moreover, we also evaluated the accuracy when the accurate estimation was determined by the set range of absolute value of the difference (e.g., within 1.5 g/dL, 2.0 g/dL) between the estimated and the actual Hb. All the detailed information on image pre-processing and the main code for this study has been provided on GitHub (https://github.com/keyan2017/hemoglobin-prediction).

### 2.7. Statistical analysis

All the statistical analysis was conducted on the R platform (R Studio, version 1.4.1717, USA). For quantitative variables, the mean, standard deviation (SD), and range are presented. For the primary effectiveness variables, 95% confidence intervals (Cis) are presented. The correlation between estimated and actual Hb was tested via Pearson analysis, wherein the r_pearson_, *P*, 95% CI were provided [ggstatsplot (18), version: 0.9.0]. Meanwhile, density distribution and scatter plots were completed with R packages [ggplot2 (19), version: 3.3.5]. All statistical tests were two-sided, and *P* < 0.05 indicated statistical significance.

## 3. Results

**Figure 2:**
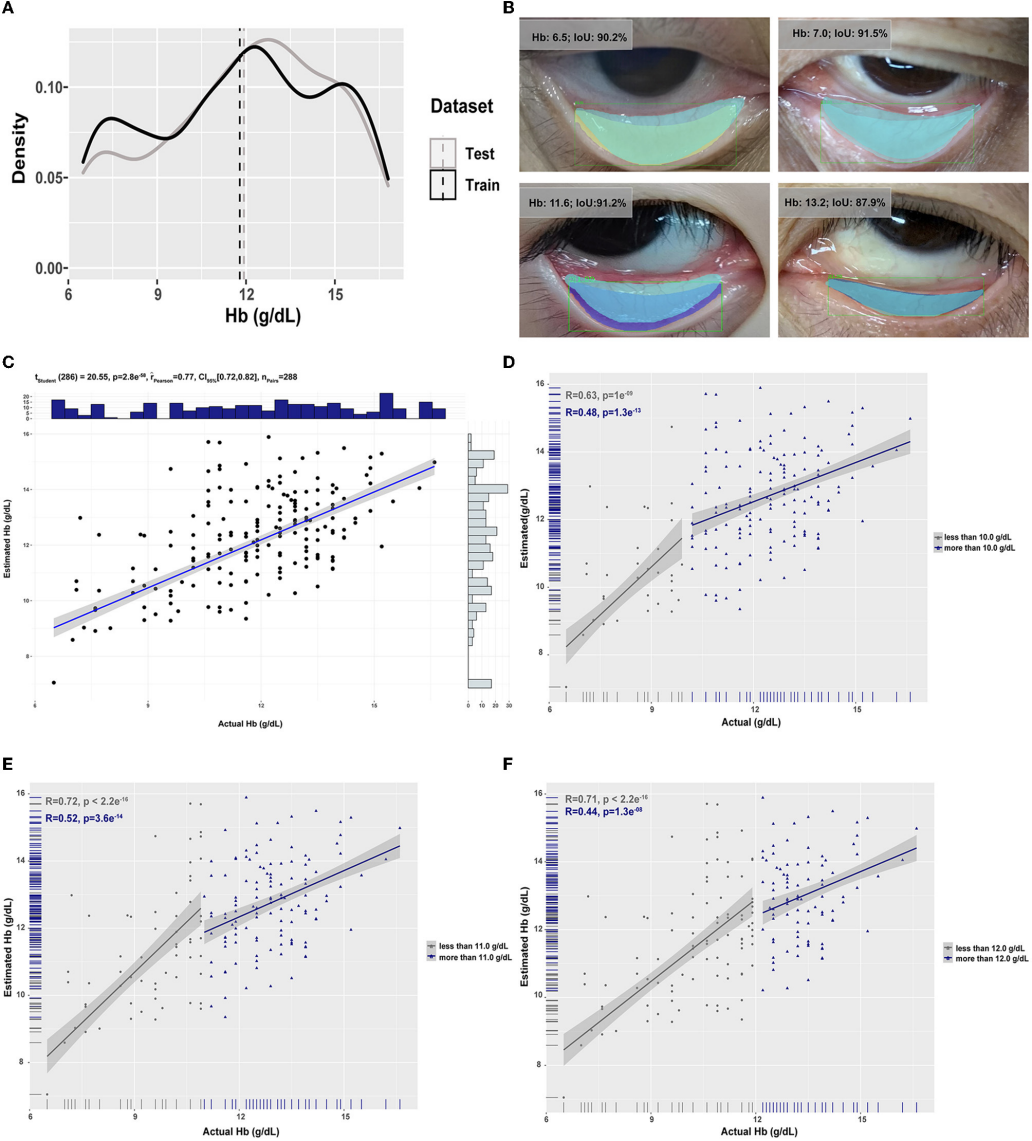
The data distribution in the training and test datasets and the performance of models are based on the combination of mask R-CNN and MobileNetV3. **(A)** The distribution of concentration of Hb in the training and test datasets. The vertical dashed lines were the mean concentration of Hb in the two datasets. **(B)** Representative overlay images of manually selected conjunctiva (light blue) and automatically recognized conjunctiva (other colors) in cases of different concentrations of Hb. The correlation between estimated and actual Hb was analyzed by Pearson analysis with different thresholds [**(C)**: no threshold; **(D)**: the threshold was 10g/dL; **(E)**: the threshold was 11 g/dL; **(F)**: the threshold was 12 g/dL].

In the current study, 1,073 pieces of eye images from 284 patients with an average age of 51.5 years old for elective surgery (M/F: 117/167) were obtained (Supplementary Table 2). Finally, 1,065 images were analyzed; three images were excluded for inadequate exposure, and five were excluded due to overexposure. After image pre-processing and up-sampling, 1,226 images were in the training dataset, and 288 were in the test dataset (Figure 1). The mean and the distribution of Hb in the training dataset were similar to those in the test dataset (Figure 2A).

Using features extracted from the manually selected palpebral conjunctiva as input to detect anemia was the most common in this area. Models directly using the manually selected palpebral conjunctiva images as input based on MobileNetV3 yielded R^2^, EVS, and MAE of 0.509 (95% CI, 0.505–0.512), 0.516 (95% CI, 0.513–0.519) and 1.6 g/dL (95% CI, 1.6–1.6), which was much better than those using features as input (Table 1). However, when the inputs were eye images, the model's performance was poorer, even based on MobileNetV3.

**Table 1:** Performance of models based on different methods with different inputs on test dataset.

**Algorithm**	**R^2^ (95% CI)**	**EVS (95% CI)**	**MAE (95% CI), g/dL**
**Models with the input of manually selected conjunctiva images**			
Decision tree regression	0.262 (0.242, 0.283)	0.267 (0.247, 0.287)	2.1 (2.0, 2.1)
Linear regression	0.300 (0.288, 0.312)	0.304 (0.292, 0.315)	2.0 (2.0, 2.0)
Support vector regression	0.267 (0.248, 0.286)	0.270 (0.252, 0.289)	2.0 (2.0, 2.0)
Gradient boosting regression	0.296 (0.283, 0.308)	0.298 (0.287, 0.31)	2.0 (2.0, 2.0)
MobileNetV3	0.509 (0.505, 0.512)	0.516 (0.513, 0.519)	1.6 (1.6, 1.6)
**Models with the input of eye images**			
Decision tree regression	−0.013 (−0.032, 0.006)	−0.001 (−0.021, 0.018)	2.4 (2.4, 2.4)
Linear regression	−0.077 (−0.113, −0.041)	−0.064 (−0.101, −0.027)	2.5 (2.4, 2.5)
Support vector regression	−0.052 (−0.081, −0.024)	−0.039 (−0.068, −0.01)	2.4 (2.4, 2.5)
Gradient boosting regression	−0.053 (−0.083, −0.024)	−0.037 (−0.067, −0.007)	2.4 (2.4, 2.5)
MobileNetV3	0.306 (0.296, 0.317)	0.338 (0.329, 0.348)	2.0 (2.0, 2.0)
**Models with the input of conjunctiva by automatically selection from eye images**			
Mask R-CNN combined with MobileNetV3	0.503 (0.499, 0.507)	0.518 (0.515, 0.522)	1.6 (1.6, 1.6)

Data were presented with 95% CIs. MAE, mean absolute error; EVS, Explained Variance Score; CI, confidence interval.

To further improve the model's performance with the eye images as input, mask R-CNN was applied for automatic segmentation of the palpebral conjunctiva from the eye images. As shown in the representative images in Figure 2B, despite the concentration of Hb (anemia or not) and the shape of the palpebral conjunctiva (wide or slender), the IoU of manually and automatically selected conjunctiva was relatively satisfied. Meanwhile, regardless of the thresholds of the mIoU, the AP and AR were relatively accepted (Table 2), which was well-matched with the existing research (4). The model based on the combination of mask R-CNN and MobileNetV3 achieved a good consequence with an R^2^ of 0.503 (95% CI, 0.499–0.507), EVS of 0.518 (95% CI, 0.515–0.522), MAE of 1.6 g/dL (95% CI, 1.6–1.6), which was similar to the model's performance using manually selected palpebral conjunctiva and was better than that directly using eye images (Table 1). The correlation between the estimated and the actual Hb was 0.77 (95% CI, 0.72–0.82); moreover, for different thresholds, the correlation between the estimated between and actual Hb remaining satisfied (Figures 2C–F). Meanwhile, when we determined the range of absolute value of the difference between the estimated and actual Hb within 2.0 g/dL as the standard of accurate estimation, the accuracy was 72.2% (Supplementary Table 3). Moreover, according to the estimated Hb, we re-evaluated the model's performance for recognizing anemia patients with different thresholds (Hb <10.0 g/dL, 11.0 g/dL, and 12.0 g/dL). When the threshold was 10.0 g/dL, the accuracy, specificity, and AUROC were 85.4%, 97.2%, and 0.752 (95% CI, 0.698–0.801) (Supplementary Table 4).

**Table 2:** The average precision and recall under different thresholds of mIoU when automatically segmentation of conjunctiva.

**Threshold of mIoU**	**AP**	**AR**	**maxDets**
0.5	0.989	0.996	100
0.5–0.95	0.672	0.720	100
0.75	0.845	0.891	100

mIoU, mask Intersection over Union; AP, average precision; AR, average recall; maxDets, maxdectetions.

## 4. Discussion

Herein, we developed a new method that could not only automatically estimate the exact concentration of Hb but also achieve a similar performance using manually selected palpebral conjunctiva as input.

As previously described, a quick and non-invasive method for Hb monitoring that can provide additional and relatively precise information between measurements of Hb using invasive blood samples is badly needed, especially for situations such as disaster rescue scenes, field rescue, emergent public health events (e.g., COVID-19), casualty transport, and battlefield rescue. Though SpHb is a non-invasive, continuous device for Hb monitoring, its application, and promotion were restricted due to its inability to be used on other platforms except Massimo's.

Numerous teams have been working on developing non-invasive methods to detect anemia or estimate the exact concentration of Hb based on computer vision technology in the last few years (Supplementary Table 1). Initially, researchers attempted to find features associated with anemia or Hb based on the manually selected palpebral conjunctiva images. The erythema index [EI = log (S_red_) – log (S_green_)], where S is the brightness of the palpebral conjunctiva in the relevant color channel) was found to be significantly associated with measured Hb (the r^2^ could be up to 0.397), based on which the sensitivity and specificity for anemia (Hb <11.0 g/dL) were 57.0 and 83.0% (20). Meanwhile, Miaou et al. (7) determined three important features, including entropy, binarization of the high Hue ratio, and PVM of G components, for detecting anemia with the palpebral conjunctiva images. Models based on these features achieved higher sensitivity and κ values than previous studies. Afterward, ANN (7, 21), Elman neural network (22), and CNN (23) were applied to detect anemia or estimate the exact concentration of Hb and achieved high accuracy. However, most of these studies were not “real” deep learning because the inputs were features extracted by feature engineering. It may be associated with the sample size being too small to fulfill the number of images needed for deep learning. However, their studies still showed that deep learning may help elevate the model's performance. Herein, we used the same method as Professor Miaou's for feature extraction and applied selected features to estimate the exact concentration of Hb using traditional regression algorithms and observed poorer performance than those directly using images as input based on MobileNetV3. It suggested that images were more informative and effective than extracted features when estimating the exact concentration of Hb. Meanwhile, deep learning algorithms may be more helpful when the inputs are images rather than features.

Despite the difference in input (features *vs*. images) and estimations (classification *vs*. regression) between previous research on models based on deep learning and ours, we compared our results with previous studies in Table 3. Though models based on MobileNetV3 with the manually selected palpebral conjunctiva achieved the best performance in the current study, the performance was much poorer when the eye images were used as input. It was suggested that the palpebral conjunctiva images as input were the most important to estimate the exact concentration of Hb or detect anemia in patients. Thus, we applied mask R-CNN to automatically segment the palpebral conjunctiva to help elevate the performance of models with eye images as input. Afterward, we got satisfactory results from segmentation, and the two-step model achieved a similar performance to that using the manually selected palpebral conjunctiva as input. Dimauro et al. (13) made great efforts to develop non-invasive and continuous Hb monitoring based on computer vision technology. In 2019, they attempted to obtain the relevant sections of the palpebral conjunctiva automatically by contour detection and feature extraction, of which the correlation between automatically extracted features and the exact concentration of Hb could be up to 0.74 (13). Recently, they attempted to apply the Biased Normalized Cuts Approach (3) and CNN (4) to automatically segment the palpebral conjunctiva from the eye images obtained using the special device and consumer-grade cameras, respectively. For images obtained using a special device, feature extraction and regression were conducted after automatic segmentation of palpebral conjunctiva, with similar results to those of manually selected palpebral conjunctiva images (3). As for the images obtained using consumer-grade cameras, the IoU score between the ground truth and the segmented mask was 85.7% (4), which is similar to ours (82.6%) (Table 3). Dimauro et al. (13) study suggested that automatically estimating the exact concentration of Hb with eye images from customer-grade cameras or smartphones is the new trend in the area of non-invasive and continuous Hb monitoring. Our results also showed that a combination of semantic segmentation and deep learning methods might be a new strategy for this area.

**Table 3:** Performance comparison between our method with previous works.

**References**	**Inputs**	**Sample Size**	**Classification/ regression/segmentation**	**Main results**
Kasiviswanathan et al. (4)	The eye images	135	Segmentation	The accuracy of the automatic segmentation was 85.7%.
Dimauro et al. (13)	The eye images	65	Segmentation	The correlation between feature “a” and Hb was 0.74.
Jain et al. (21)	The conjunctiva images	99	Classification	The accuracy, sensitivity and specificity for prediction anemia was 97.00%, 99.21% and 95.42%.
Saldivar-Espinoza et al. (23)	The conjunctiva images	300	Classification	The Sensitivity, accuracy, and specificity were 77.6%, 43.0%, and 36.0%.
Muthalagu (22)	The conjunctiva images	127	Classification	The sensitivity and specificity for detecting anemia were 77.3% and 96.1%.
Collings et al. (20)	The conjunctiva images	94	Classification	The sensitivity and specificity were 57.0% and 83% in the internal validation datasets.
Our method	The eye images	1065	Segmentation	The accuracy of the automatic segmentation was 82.6%.
			Regression	The correlation, MAE between the estimated and the actual Hb was 0.77, 1.6 g/dL. The accuracy, specificity, and AUROC were 85.4%, 97.2%, and 0.752 (Hb threshold was 10.0 g/dL)

Hb, hemoglobin; MAE, mean absolute error; AUROC, area under the receiver operating characteristic curve.

Our method was more convenient and simpler than previous ones since manually selecting the conjunctiva is no longer needed before inputting the images. There were some other advantages to our study. First of all, the sample size of the original images was larger compared with previous research (Supplementary Table 1), which would reduce overfitting and increase robustness. Second, smartphones obtained images when patients were lying on their backs awake or anesthetized, which would be more convenient for promotion and application in various situations. Third, herein, we estimated the exact concentration of Hb, which was seldom conducted in previous studies. Estimating the exact concentration of Hb could not only indicate the trend change of Hb but also easily detect anemia according to various thresholds without repeated image labeling (Supplementary Table 3). In summary, the combination of mask RCNN and MobileNetV3 to automatically estimate the exact concentration of Hb is quite promising in the area of non-invasive and continuous Hb monitoring in a variety of situations.

There are some limitations to the current study. First, though we tried to enroll more images for analysis and model development, the amounts of images from anemia and non-anemia were still imbalanced. The model's performance might be better if more images were enrolled, especially those from patients with anemia. Second, the images were obtained from one center, so external validation was not conducted. Multicenter research should be conducted to further increase the model's performance and robustness. Third, there is a significant difference in the mean Hb concentration between the Hb level from ABG and the standard venous analyzers, so images labeled with Hb measured by the standard venous analyzers should be enrolled to correct bias.

## 5. Conclusion

In summary, we developed a method to estimate the exact concentration of Hb based on a combination model of mask R-CNN and MobileNetV3, which achieved an R^2^ of 0.503 (95% CI, 0.499–0.507) and an MAE of 1.6 g/dL (95% CI, 1.6–1.6). It can help medical staff's decision-making with high efficiency, especially in disaster rescue scenes, field rescue, emergent public health events, casualty transport, and battlefield rescue. Furthermore, our method was more convenient and simpler than previous ones since manually selecting the conjunctiva is no longer needed before inputting the images.

## Data availability statement

The original contributions presented in the study are included in the article/Supplementary material, further inquiries can be directed to the corresponding authors.

## Ethics statement

The studies involving human participants were reviewed and approved by the Institutional Ethics Committee of the First Affiliated Hospital of Third Military Medical University (KY2021060). The patients/participants provided their written informed consent to participate in this study.

## Author contributions

BY and Y-wC supervised the study designation, analysis, and manuscript edits. X-yH performed data acquisition, image processing, and manuscript drafting. Y-jL performed study designation, statistical analysis, and manuscript drafting. XS and Y-zS acquired data and screened samples. A-lS made implementation, figure creation, and manuscript edits. HL has made data acquisition, sample screening, and manuscript edits. Y-sL has made contributions to enroll participants and sign informed consent. X-fW has made contributions to image processing and obtained funding. L-fT performed the acquisition and interpretation of the data. C-yY made contributions to the discussion of study designation and data acquisition. Z-yY performed data analysis and drafted the manuscript. L-qX contributed to the technical, implementation, figure creation, and manuscript edits. All authors contributed to the article and approved the submitted version.
