# Supplementary material for: A new, feasible, and convenient method based on semantic segmentation and deep learning for hemoglobin monitoring
Source: Front Med (Lausanne). 2023 Aug 3;10:1151996. doi: 10.3389/fmed.2023.1151996 (PMC10435289; doi:10.3389/fmed.2023.1151996)
Supplement: Supplementary file 1 [file Data_Sheet_1.docx]

Supplementary Material

A new, feasible and convenient method based on semantic segmentation and deep learning for hemoglobin monitoring

Xiao-yan Hu^1^†, Yu-jie Li^1^†, Xin Shu^1^, Ai-lin Song^1^, Hao Liang^1^, Yi-zhu Sun^1^, Xian-feng Wu^1^, Yong-shuai Li^1^, Li-fang Tan^1^, Zhi-yong Yang^1^, Chun-yong Yang^1^, Lin-quan Xu^2^, Yu-wen Chen^2*^, Bin Yi^1*^

**†** These authors contributed equally to this work and share the first authorship

* Correspondence: Bin Yi: yibin1974@163.com; Yu-wen Chen: [chenyuwen@cigit.ac.cn](mailto:chenyuwen@cigit.ac.cn).

# Supplementary methods

**The criteria of image cutting and labelling**

The selected half-face images were cut as eye images following the criteria: The range of eye images included the highest point of upper eyelid, the lowest point of lower eyelid, the inner and outer canthus (shown in supplementary Fig.1 A). The labelling of conjunctiva from the eye images followed the criteria: label the palpebral conjunctiva as more as possible, meanwhile avoid the area of optic papilla and bulbar conjunctiva (shown in supplementary Fig.1 B-C).


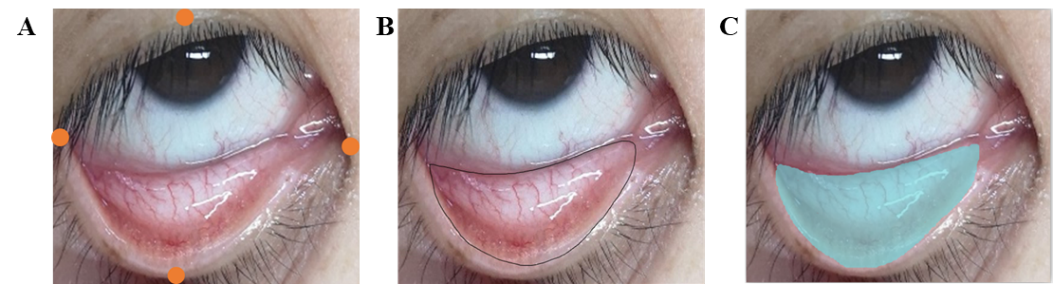


Supplementary Figure **1.** The criteria of image cutting and labelling. (A) The criteria of cutting eye images from the half-face images. The criteria of labelling of conjunctiva from eye images by photoshop (B) and Colabeler (C). The circles filled with orange were the four dentification points for image cutting from half-face images.

**The detailed architecture of Mask R-CNN and MobileNetV3**

This is a two-step method based on combination of Mask R-CNN and MobileNetV3. As shown in supplementary Fig.2, pre-processed images were inputted into the pre-trained ResNet+FPN to get the feature map; In turn, the feature map obtained ROI by Regional Proposal Network (RPN); Binary classification of foreground and background was conducted by sigmoid classifier, more accurate candidate bounding box position information was also obtained by bounding box regression; Furthermore, part of ROI was filtered out under non-maximum suppression. Afterwards, the feature map and the last remaining ROI were sent to the ROI Align layer, enabling each ROI to generate a fixed-size feature map. Finally, the flow passes through two branches, one branch enters the fully connected layer for object classification and frame regression, and the other branch enters the full convolutional network (FCN) for pixel segmentation ([1](#_ENREF_1)).

MobileNetV3 was proposed by Howard et al based on MobileNetV2 and MobileNetV1, using network architecture search (NAS)([2](#_ENREF_2)). The MobileNetV3 block contains a core building block called the inverted residual block, which includes a depthwise separable convolution block and a squeeze-and-excitation (SE) block. The depthwise separable convolutional is used to alter the traditional convolution block and reduce the model capacity. The SE block is used to pay more attention to the relevant features on each channel during training.


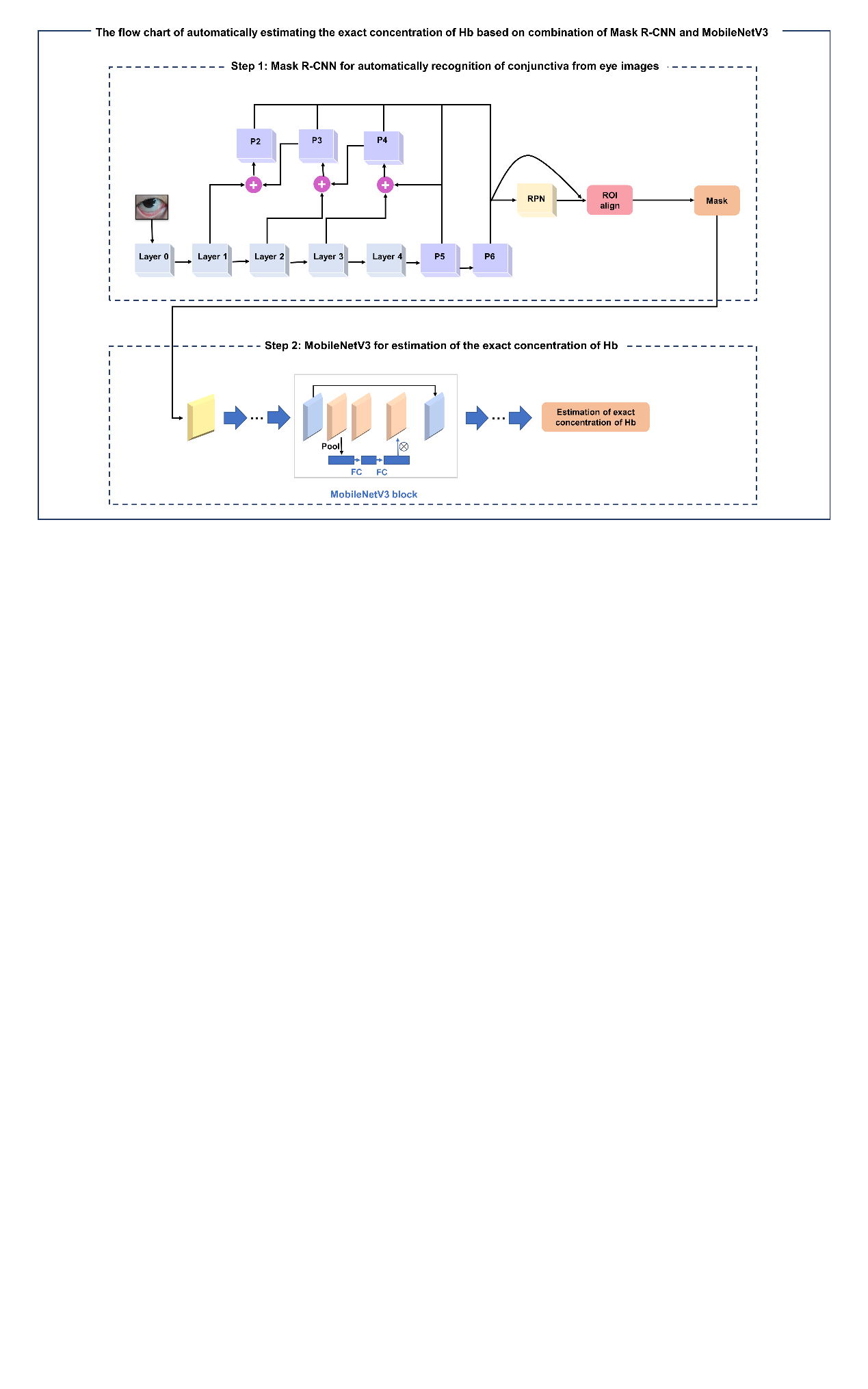


**Supplementary Figure 2.** The flow chart of automatically estimating the exact concentration of Hb based on combination of Mask R-CNN and MobileNetV3. RPN, Regional Proposal Network; ROI, region of interest; FC, Full connection.

Supplementary Table 1. Summary of the associated research.

| **Reference** | **Sample Size** | **Equipment for image acquisition** | **Inputs** | **Method** | **Main results** |
| --- | --- | --- | --- | --- | --- |
| Selim Suner, et al. J Emerg Med. 2007 ([3](#_ENREF_3)) | 117 images | - Digital camera - 18% standard grayscale card for calibration color | Part of the palpebral conjunctiva | Not mentioned (Features were R, G, B) | Threshold :11.0 g/dL   - The sensitivity and specificity were 69.0% and 72.0%. - The correlation between estimated and actual Hb was 0.522 by Pearson analysis. |
| Chen Y, M.et al. Comput Methods Programs Biomed. 2016 ([4](#_ENREF_4)) | 100 images | - Digital camera | Part of the palpebral conjunctiva | Q (Features were HHR, PVM); SVM and ANN (Features were entropy, binarization of HHR, and PVM of G component) | Threshold :11.0 g/dL   - Q: The kappa value, sensitivity and specificity were 0.53, 62.0%, 90.0%. - ANN: The kappa value, sensitivity and specificity were 0.58, 78.0%, and 75.0%. - SVM: The kappa value, sensitivity and specificity were 0.61, 75.0%, and 83.0%. |
| Shaun Collings, et al. Plos One. 2016 ([5](#_ENREF_5)) | 94 images | - Digital camera and smartphone - Color calibration card | The manually selected palpebral conjunctiva | Feature engineering (The extracted feature was EI) | Threshold :11.0 g/dL   - The performance of models based on EI (with an accuracy of 70.0% and 72.0% based on images obtained by LX5 and iPhone) is better than that based on clinician assessment (the accuracy of three clinicians were 60.0%, 57.0% and 64.0%) - For detection of anemia patients by feature engineering, the sensitivity and specificity were 57.0% and 83% in the internal validation datasets. |
| Tamir A, et al. IEEE Region 10 Humanitarian Technology Conference (R10-HTC), 2017 ([6](#_ENREF_6)) | 19 images | - Smartphone - Ambient lighting condition | The manually selected palpebral conjunctiva | The extracted feature was red and green pixels from RGB color space | Threshold: diagnostic criteria of anemia   - 15 out of the 19 cases predicted successful (accuracy 78.9%). |
| Vitoantonio Bevilacqua, et al. 2016 IEEE International Symposium on Medical Measurements and Applications (MeMeA). 2016 ([7](#_ENREF_7)) | 77 images | - Smartphone - Acquisition device | The manually selected palpebral conjunctiva | SVM (extracted feature was “a” from Lab color space) | Threshold: diagnostic criteria of anemia   - The correlation between the feature “a” and Hb was 0.56 by Pearson analysis. - The accuracy, specificity, and sensitivity were84.4%, 82.4%, and 100%, respectively. |
| Anggraeni MD, et al. Materials Science and Engineering. 2017 ([8](#_ENREF_8)) | 20 images | - Smartphone - White paper | The manually selected palpebral conjunctiva | LR (extracted feature was “R” from RGB color space) | Threshold :11.0 g/dL   - The correlation between “R” color intensity and Hb were 0.814. |
| Yi-Ming Chen et al. Journal of Healthcare Engineering. 2017 ([9](#_ENREF_9)) | 100 images | - Digital camera | Part of the palpebral conjunctiva | KF+NPR (extracted feature was “R” from RGB color space) | Threshold :11.0 g/dL   - The sensitivity and specificity were 76.2% and 80.9%. |
| R.Muthalagu, et al. TAGA JOURNAL. 2018 ([10](#_ENREF_10)) | 127 images | - iPhone - Different conditions of lighting | The manually selected palpebral conjunctiva | ANN (extracted feature was Hue, Saturation, Intensity from HIS color space) | Threshold :10.0 g/dL   - The sensitivity and specificity for detecting anemia were 77.3% and 96.1% |
| Gerson Delgado-Rivera, et al. IEEE International Conference on Automation. 2018 ([11](#_ENREF_11)) | 115 images | - Smartphone | The eye images | automatic segmentation (extracted feature was “R” from RGB color space) | - The accuracy of the automatic segmentation was 92.2% |
| Giovanni Dimauro, et al. IEEE Access. 2018 ([12](#_ENREF_12)) | 113 images | - Smartphone - Special device | The manually selected palpebral conjunctiva | KNN (extracted feature were a, b, and the G value from the RGB color space) | Threshold: high-risk (Hb < 10.5 g/dL), doubtful (10.5 g/dL < Hb < 11.5 g/dL), low-risk (Hb > 11.5 g/dL)   - accuracy 100% - Correlation between feature “a” and Hb was 0.745 by Pearson analysis. |
| Bryan Saldivar-Espinoza, et al. Neural Information Processing Systems. 2019 ([13](#_ENREF_13)) | 300 images | - Smartphone - Color card | The manually selected palpebral conjunctiva | CNN (extracted feature were R and G from RGB color space) | Threshold:11.0 g/dL   - The Sensitivity, accuracy, and specificity were 77.6%, 43.0%, and 36.0% at the best cut-off. |
| Giovanni Dimauro, et al. SpliTech. 2019 ([14](#_ENREF_14)) | 65 images | - Smartphone - Special device | The eye images | automatic segmentation (extracted feature were “a” from Lab color space) | - The correlation between feature “a” and Hb was 0.74. |
| Giovanni Dimauro, et al. IEEE Access. 2019 ([15](#_ENREF_15)) | - 102 original images - 708 images after SMOTE up-sampling - 354 images after ROSE up-sampling | - Smartphone - Special device | The manually selected palpebral conjunctiva | SLIC Superpixe in segmentation;  SMOTE-kNN /ROSE-kNN for estimation | Threshold :11.5 g/dL   - The accuracy, sensitivity and specificity of the original unbalanced dataset were 76.5%, 56.0%, and 83.1%. - The accuracy, sensitivity and specificity of models based on Smote-kNN were 98.2%, 99.5% and 96.4% (n=708). - The accuracy, sensitivity and specificity of models based on ROSE-kNN were 98.0%, 100.0%, and 96.3% (n=354) |
| Prakhar Jain, et al. Int J Imaging Syst Technol. 2019 ([16](#_ENREF_16)) | - 99 images - 3103 images after augmentation | - Smartphone | The manually selected palpebral conjunctiva | ANN (extracted feature were R from RGB color space) | Threshold: not mentioned   - The accuracy, sensitivity and specificity for prediction anemia was 97.00%, 99.21% and 95.42% |
| Nahiyan Bin Noor, et al. BECITHCON. 2019 ([17](#_ENREF_17)) | 104 images | - Smartphone - Special device | The manually selected palpebral conjunctiva | DT/SVM/KNN (feature were EI and “a” from lab color space) | Threshold :10.0 g/dL   - For Anemia detection, the accuracy of DT, SVM, and KNN was 82.61%, 73.91%, and 73.91%, respectively. |
| Sivachandar Kasiviswanathan, et al. Electronics. 2020 ([18](#_ENREF_18)) | 135 images | - Smartphone - Special device | The eye images | UNBCSM (no feature selection) | - IoU score between ground truth and the segmented mask was 85.7% for validation dataset - The performance of model based on manual selected conjunctiva images was similar to that with automatic segmentation conjunctiva. |
| Giovanni Dimauro et al. Electronics. 2020 ([19](#_ENREF_19)) | 94 images | - Camera - Special device | The eye images | Semantic segmentation | - The F1-measure between manually segmentation and automatically segmentation was 0.904, the accuracy, sensitivity and specificity were 96.41%, 86.73% and 94.63%. |

Hb, hemoglobin; Q, quick algorithm; SVM, support vector machine; ANN, artificial neural network; HHR, High Hue Rate; R, Red Color Intensity; PVM, pixel value in the middle; EI, erythema index; LR, linear regression; KF, Kalman filter; NPR, Nonlinear Penalty Regression; k-NN, k-nearest neighbor; DT, decision tree; CNN, Convolutional Neural Network; PCC, Pearson correlation coefficient; SLIC, simple linear iterative clustering; SMOTE, Synthetic Minority Over-Sampling Examples; ROSE, Random Over-Sampling Examples; UNBCSM, U-Net Based Conjunctiva Segmentation Model.

# Supplementary results

**Supplementary Table 2.** **Basic information of the enrolled patients.**

| **Variables** |  |
| --- | --- |
| **Age (mean ± standard deviation)** | 51.5 ± 12.1 |
| **Male [n (%)]** | 117 (41.1) |
| **Female [n (%)]** | 167 (58.8) |
| **Classification of main diagnosis [n (%)]** |  |
| Urinary system diseases | 27 (9.5) |
| Obstetrics and Gynecology system diseases | 53 (18.7) |
| Hepatobiliary and pancreatic system diseases | 102 (35.9) |
| Gastrointestinal system diseases | 46 (16.2) |
| Spinal and limb system diseases | 22 (7.7) |
| Craniocerebral system diseases | 12 (4.2) |
| Cardiothoracic system diseases | 22 (7.7) |

**The performance of models based on combination of mask R-CNN and MobileNetV3 to detection of anemia at different thresholds.**

Due to that most of the previous researches were for detecting anemia at a set threshold of Hb, we tried to evaluate the performance for detection of anemia at different thresholds. In the current study, we determined the estimated classification of anemia by results estimated concentration of Hb, then confusion matrix was calculated. Afterwards, the sensitivity, specificity, accuracy, and area under the receiver operating characteristic curve (AUROC) were applied for evaluation. Herein, concentration of Hb less than 10.0 g/dL, 11.0 g/dL and 12.0 g/dL were selected as thresholds.

When we determined the range of absolute value of the difference between the estimated and actual Hb within 2.0 g/dL as the standard of accurate estimation, the accuracy was 72.2% (supplementary Tab.3).

**Supplementary Table 3.** **The accuracy of the new method at various thresholds of the difference between the estimated and actual Hb.**

| **Absolute value of the difference between the estimated and actual Hb** | **Accuracy** |
| --- | --- |
| **within 1.5 g dL^-1^** | 52.4% |
| **within 2.0 g dL^-1^** | 72.2% |
| **within 2.5 g dL^-1^** | 83.7% |

Herein, thresholds of 11.0 g/dL and 12.0 g/dL were selected for comparisons with previous works, and 10.0 g/dL was selected for the decision-making of blood transfusion. For the threshold of 11.0 g/dL, the sensitivity and specificity of detection of anemia were 78.1% and 75.4%, which were similar to the previous works. Moreover, for the threshold of 10g/dL, the sensitivity, specificity, and accuracy were 53.2%, 97.2% and 85.4%. In summary, performance of the model based on combination of mask R-CNN and MobileNetV3 was satisfied for detection of anemia at different thresholds with the advantages of convenience and easy to promotion.

**Supplementary Table 4** **The model performance of combined algorithm based on different threshold.**

| **Threshold** | **Sensitivity (%)** | **Specificity (%)** | **Accuracy (%)** | **AUROC** |
| --- | --- | --- | --- | --- |
| **10.0 g/dL** | 53.2 (41.5 - 64.7) | 97.2 (93.9 - 98.9) | 85.4 | 0.752 (0.698 -0.801) |
| **11.0 g/dL** | 78.1 (69.0 - 85.6) | 75.4 (68.5 - 81.5) | 76.4 | 0.768 (0.714-0.815) |
| **12.0 g/dL** | 71.0 (62.7 - 78.4) | 80.7 (73.4 - 86.7) | 76.0 | 0.758 (0.705-0.807) |

AUROC, area under the receiver operating characteristic curve.

**REFERENCES**

1. Kaiming He GG, Piotr Dollár, Ross Girshick. mask R-CNN. IEEE International Conference on Computer Vision (ICCV): IEEE; 2017. doi:10.1109/ICCV.2017.322

2. Andrew Howard MS, Grace Chu, Liang-Chieh Chen, Bo Chen, Mingxing Tan, Weijun Wang, et al. Searching for MobileNetV3. 2019 IEEE/CVF International Conference on Computer Vision (ICCV); 2019.

3. Suner S, Crawford G, McMurdy J, Jay G. Non-invasive determination of hemoglobin by digital photography of palpebral conjunctiva. The Journal of emergency medicine. 2007;33(2):105-11. doi:10.1016/j.jemermed.2007.02.011

4. Chen YM, Miaou SG, Bian H. Examining palpebral conjunctiva for anemia assessment with image processing methods. Computer methods and programs in biomedicine. 2016;137:125-35. doi:10.1016/j.cmpb.2016.08.025

5. Shaun Collings, Oliver Thompson, Evan Hirst, Louise Goossens, Anup George, Robert Weinkove. Non-Invasive Detection of Anaemia Using Digital Photographs of the Conjunctiva. Plos One. 2016;10. doi:10.1371/journal.pone.0153286 A

6. Tamir A, Jahan CS, Saif MS, Zaman SU, Islam MM, Khan AI, et al. Detection of anemia from image of the anterior conjunctiva of the eye by image processing and thresholding. 2017 IEEE Region 10 Humanitarian Technology Conference (R10-HTC); Dhaka, Bangladesh2017. p. 697-701. doi:10.1109/r10-htc.2017.8289053

7. Vitoantonio Bevilacqua GD, Francescomaria Marino, Antonio Brunetti FC, Antonio Di Maio, Enrico Nasca, Gianpaolo Francesco Trotta, et al. A novel approach to evaluate blood parameters using computer vision techniques. 2016 IEEE International Symposium on Medical Measurements and Applications (MeMeA); 2016.

8. M D Anggraeni AF. Non-invasive Self-Care Anemia Detection during Pregnancy Using a Smartphone Camera. Materials Science and Engineering. 2017. doi:10.1088/1757-899X/172/1/012030

9. Chen YM, Miaou SG. A Kalman Filtering and Nonlinear Penalty Regression Approach for Noninvasive Anemia Detection with Palpebral Conjunctiva Images. Journal of healthcare engineering. 2017;2017. doi:10.1155/2017/9580385

10. R.Muthalagu VTBaSJ. A Smart (phone) Solution: An effective tool for Screening Anaemia - Correlation with conjunctiva pallor and haemoglobin levels. TAGA JOURNAL. 2018;14:2611-21.

11. Gerson Delgado-Rivera AR-G, Alicia Alva-Mantari, Bryan Saldivar-Espinoza, Mirko Zimic, Franklin Barrientos-Porras, Mario Salguedo-Bohorquez. Method for the Automatic Segmentation of the Palpebral Conjunctiva using Image Processing. IEEE International Conference on Automation/XXIII Congress of the Chilean Association of Automatic Control (ICA-ACCA): IEEE; 2018. doi: 10.1109/ICA-ACCA.2018.8609744

12. Dimauro G, Caivano D, Girardi F. A New Method and a Non-Invasive Device to Estimate Anemia Based on Digital Images of the Conjunctiva. Ieee Access. 2018;6:46968-75. doi:10.1109/access.2018.2867110

13. Bryan Saldivar-Espinoza1 DN-F, Franklin Porras-Barrientos1, Alicia Alva-Mantari1 LSL, Mirko Zimic. Portable system for the prediction of anemia based on the ocular conjunctiva using Artificial Intelligence. Neural Information Processing Systems (NeurIPS 2019). 2019;3.

14. G Dimauro LB, D Caivano, G Colucci, F Girardi Automatic segmentation of relevant sections of the conjunctiva for non-invasive anemia detection. 2018 3rd International Conference on Smart and Sustainable Technologies (SpliTech) 2019/10/26 2019.

15. Dimauro G, Guarini A, Caivano D, Girardi F, Pasciolla C, Iacobazzi A. Detecting Clinical Signs of Anaemia From Digital Images of the Palpebral Conjunctiva. Ieee Access. 2019;7:113488-98. doi:10.1109/Access.2019.2932274

16. Jain P, Bauskar S, Gyanchandani M. Neural network based non‐invasive method to detect anemia from images of eye conjunctiva. International Journal of Imaging Systems and Technology. 2019;30(1):112-25. doi:10.1002/ima.22359

17. Noor NB, Anwar MS, Dey M. Comparative Study Between Decision Tree, SVM and KNN to Predict Anaemic Condition. Biomedical Engineering, Computer and Information Technology for Health (BECITHCON)2019. p. 24-8. doi:10.1109/BECITHCON48839.2019.9063188

18. Kasiviswanathan S, Bai Vijayan T, Simone L, Dimauro G. Semantic Segmentation of Conjunctiva Region for Non-Invasive Anemia Detection Applications. Electronics. 2020;9(8):1309. doi:10.3390/electronics9081309

19. Dimauro G, Simone L. Novel Biased Normalized Cuts Approach for the Automatic Segmentation of the Conjunctiva. Electronics. 2020;9(6):997. doi:10.3390/electronics9060997
